# Weakly electric fish use self-generated motion to discriminate object shape

**DOI:** 10.1016/j.anbehav.2023.08.002

**Published:** 2023-11

**Authors:** Sarah Skeels, Gerhard von der Emde, Theresa Burt de Perera

**Affiliations:** a Department of Biology, University of Oxford, Oxford, U.K.; b Institute of Zoology, University of Bonn, Bonn, Germany

**Keywords:** active electrolocation, active sensing, body movement, *Gnathonemus petersii*, object recognition, sensory-motor integration, shape discrimination, weakly electric fish

## Abstract

Body movements are known to play an active role in sensing. However, it is not fully understood what information is provided by these movements. The Peter's elephantnose fish, *Gnathonemus petersii*, sense their environment through active electrolocation during which they use epidermal electroreceptors to perceive object-induced distortions of a self-produced electric field. The analysis of electric images projected on their skin enables them to discriminate between three-dimensional objects. While we know the electric image parameters used to encode numerous object properties, we do not understand how these images encode object shape. We hypothesized that ‘movement-induced modulations’ (MIMs) elicited by body movements might be involved in shape discrimination during active electrolocation. To test this, we trained fish to complete a shape discrimination task in a two-alternative forced-choice set-up, and then manipulated the space available to individuals for scanning movements to see whether this led to a change in their discrimination performance. We found that if enough space was available, fish were very good at discriminating objects of different shapes. However, performance decreased when the space was reduced so that scanning movements were impaired. Our study demonstrates the importance of body movements for gaining complex environmental information such as object shape through active electrolocation. Movement can enhance perception by allowing the extraction of certain kinds of information. Similar observations have been made in other animals using different senses, suggesting that the core principles of sensory-motor integration might be valid for various sensory modalities.

## Highlights

• Animals can move their bodies to improve perception of their environment.
• *G. petersii* can use movement for shape recognition during active electrolocation.
• Discrimination performance worsened as space around the objects was restricted.
• Response times and movement behaviour also changed as space was reduced.
• Parallels with active sensing strategies observed in animals with different senses.

Animals can move their bodies to improve perception of their environment (see more detailed discussions in Hofmann, Sanguinetti-Scheck, Künzel, et al., 2013; Skeels et al., 2023a). These ‘active sensing strategies’ are observed across many taxa and in a range of sensory systems, from active vision in insects (e.g. Voss & Zeil, 1998) to echolocation in bats (e.g. Yin & Müller, 2019). The fact that these strategies are recurring highlights their fundamental importance for optimizing and shaping sensory input (see reviews by Hofmann, Sanguinetti-Scheck, Künzel, et al., 2013; Skeels et al., 2023a). This is probably best understood in active vision, where head and body movements can modify visual perception in a number of ways (see reviews by Land, 1999; Kral, 2003). For example, Parker et al. (2022) demonstrated that laboratory mice can successfully judge distances when jumping to platforms even under monocular conditions (when vision in one eye was obscured). The mice increased their head movements and altered their head position when completing the task with monocular rather than binocular vision (Parker et al., 2022). It is likely that these movements helped them to successfully estimate the distance of gaps through motion or positional parallax which requires temporal integration of information (Parker et al., 2022). Echolocating bats are also known to change their movements to improve sensing. Recently, Taub and Yovel (2020) demonstrated that bats, *Pipistrellus kuhlii*, can alter their flight movements during echolocation to help them separate desired signals (e.g. of prey targets) from a noisy environmental background. Individuals were shown to approach their target from a smaller angle of attack, which resulted in weaker echoes (and consequently less noise from the background) meaning that target signals could be detected better. These examples illustrate on a general level that movement can be important for sensory acquisition in a wide range of animals and senses. Although the exact function and mechanism underpinning these behaviours will likely vary, the general point remains that movement seems to be important in enhancing sensing in these animals.

**Figure 1:**
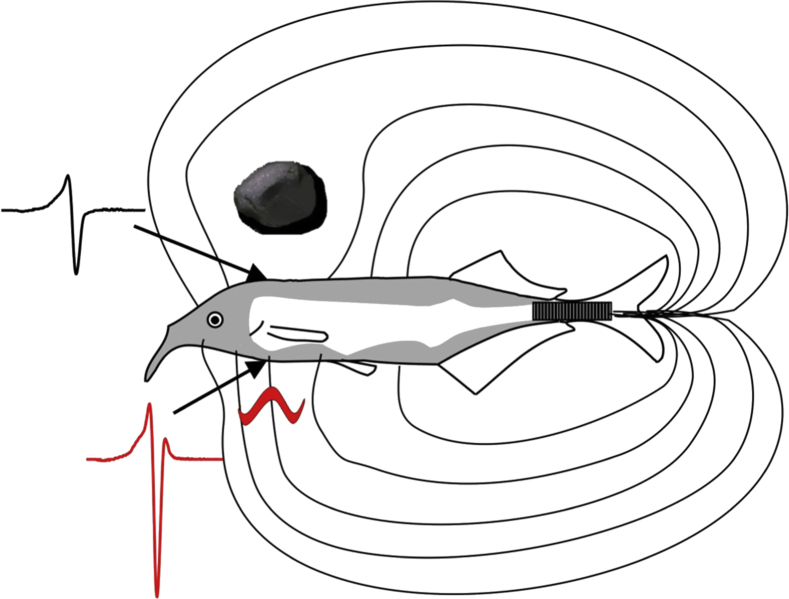
*Gnathonemus petersii* using their active electric sense to investigate nearby objects. The electric organ, found in the caudal peduncle (black rectangle), generates electric organ discharges (EODs). Each EOD generates a three-dimensional electric field around the fish (shown by the contour lines). Objects within the electric field will distort it according to their intrinsic properties. For example, the worm (shown in red) is an electrical conductor, so it will increase the amplitude and modify the waveform of local EODs. It will also increase field line density. On the other hand, the rock (shown in black) is a nonconductor, so it will decrease the amplitude of local EODs, but the waveforms will remain unchanged. Field line density will decrease as well. These changes are detected by epidermal electroreceptors (shown in grey). The information is then sent to the brain for processing. Diagram reproduced with permission from von der Emde (2006).

Weakly electric fish also seem to use movement to modify their perception (reviewed by Hofmann, Sanguinetti-Scheck, Künzel, et al., 2013; Skeels et al., 2023a). Weakly electric fish, such as *Gnathonemus petersii* (Mormyridae)*,* possess an active electric sense that they use to detect objects in their environment, termed active electrolocation (Lissmann, 1951, 1958; Lissmann & Machin, 1958; Heiligenberg, 1973; Fig. 1). These fish generate electric organ discharges (EODs) from their electric organ (Fig. 1). Each EOD produces a three-dimensional electric field around the fish (Fig. 1). Objects within this field close to the fish's skin (on the centimetre range) will distort it, and specialized electroreceptors on the skin detect these changes (reviewed in von der Emde, 2006; von der Emde & Zeymer, 2020; Fig. 1). The area of change on the skin is referred to as ‘the electric image’ of the object (Caputi & Budelli, 2006; von der Emde, 2006).

In many early training experiments examining active electrolocation in *G. petersii*, the EODs were recorded, and their patterns described (e.g. von der Emde, 1992; von der Emde & Fetz, 2007). These fish constantly produce EODs, but they can alter the rate according to the context. von der Emde and Fetz (2007) found that *G. petersii* would transiently regularize and increase their EOD rate (up to 70 Hz) when approaching and actively inspecting an object during a recognition task as they obtained information on that object. This rate increased to 80 Hz when they passed by the gate with the object behind it (von der Emde & Fetz, 2007). However, when freely swimming in their living compartment, the EOD rate was much lower and irregular (von der Emde & Fetz, 2007). EOD production is often energetically expensive; therefore, individuals will reduce their EOD rate when they no longer need to sample an object (Markham et al., 2016).

The active electric sense is well suited to object recognition, as it provides fine-scale spatial information of objects less than about half a fish length away (von der Emde et al., 2010; Schumacher, Burt de Perera, Thenert, & von der Emde, 2016; Schumacher, Burt de Perera, & von der Emde, 2017, 2017b). Individuals can determine object properties by examining different image parameters within an electric image, such as amplitude distribution within the image, local waveform distortion and image size (von der Emde, 2006; von der Emde & Fetz, 2007; von der Emde & Zeymer, 2020). Thus, by analysing single electric images (‘snapshots’), it is possible for the fish to get information about an object's size, material composition, distance and electric colour (von der Emde et al., 1998; von der Emde & Fetz, 2007; Budelli & Caputi, 2000; Gottwald et al., 2017, 2018). For example, distance can be calculated by taking the ratio between the maximal slope and maximum amplitude of a single electric image (von der Emde et al., 1998). On the other hand, electric colour is based on the ratio between the amplitude and waveform modulations of an electric image (Budelli & Caputi, 2000; Gottwald et al., 2017, 2018). It provides a fast and reliable cue for detecting prey, as different living entities will be associated with different electric colours, and is independent of size, distance and position (Gottwald et al., 2018).

However, no parameter or collection of parameters in a single electric image can explain recognition of three-dimensional object shape (von der Emde, 2004, 2006; Schumacher, Burt de Perera, & von der Emde, 2016; Fujita & Kashimori, 2019). Shape is a complex cue to extract from the environment, since it is made up of a collection of features that all contribute towards an object's overall shape (e.g. object height, presence or absence of corners, the number, length and orientation of sides; von der Emde & Fetz, 2007). Moreover, the perception of shape during electrolocation is, like in other senses, viewpoint dependent (Fujita & Kashimori, 2019; Schumacher, Burt de Perera, & von der Emde, 2016). Additionally, the shape of an object at a given viewpoint cannot be taken as the shape of the corresponding electric image. This is because the electric image will change shape according to the body region onto which it is projected (Pusch et al., 2008). For example, a cube will have a much steeper electric image slope when facing a flat surface, such as the body trunk, than when positioned close to a curved sensory surface, like the head (Pusch et al., 2008). This is largely due to the ‘edge effect’ (Sicardi et al., 2000) which describes how the current is drained and is related to both the geometry of the object but also the geometry of the receptive surface (Pusch et al., 2008). There is also no such mechanism within the electrosensory system to focus the electric image and the asymmetric nature of the electric field generated around the fish means that the electric image generated will always be distorted (Caputi & Budelli, 2006; von der Emde, 2006; Engelmann et al., 2008; Pusch et al., 2008). As such, there is no clear geometric relationship between the actual shape of an object and single electric images (Caputi & Budelli, 2006; von der Emde, 2006).

Nevertheless, we know that *G. petersii* can recognize three-dimensional shapes and accurately discriminate between them (von der Emde & Schwarz, 2000, 2002; von der Emde, 2004; von der Emde & Fetz, 2007; von der Emde et al., 2010; Schumacher, Burt de Perera, Thenert, & von der Emde, 2016; Schumacher, Burt de Perera, & von der Emde, 2016*,* 2017; Skeels, 2022). They seem to use features within an object to recognize and generalize its shape, even when the object is rotated (von der Emde & Fetz, 2007; von der Emde et al., 2010; Schumacher, Burt de Perera, & von der Emde, 2017; Skeels, 2022; Skeels et al., 2023b). In the wild, it would be advantageous for *G. petersii* to determine an object's shape accurately, for example for landmark recognition. During the day, mormyrids are often found under tree roots or dense vegetation (Francke et al., 2014; Moller et al., 1979; Tawari-Fufeyin & Ekaye, 2007). However, at night, they will leave these fixed rest sites to forage, often travelling many tens of metres (Moller et al., 1979). To navigate to and from their rest site reliably, an individual may use local landmarks along their routes, such as distinctive large rocks or submerged tree roots. Shape might be a useful feature in identifying these objects reliably.

The ability to discriminate shape might help an individual to compensate for ‘electric illusions’ which can develop when assessing the distance of certain objects (von der Emde & Schwarz, 2000; Schwarz & von der Emde, 2001). *Gnathonemus petersii* have difficulty estimating the distance of spheres, often judging them to be further away than other objects placed at the same distance (von der Emde et al., 1998). This might be the result of spheres producing smaller slope to amplitude ratios than other objects of equivalent distance, presumably because of their curvature (von der Emde et al., 1998). However, fish trained for a prolonged time with a variety of objects no longer made these mistakes, perhaps because they were able to recognize the shapes of the objects and then take into account the errors associated with estimating their distances (von der Emde & Schwarz, 2000; Schwarz & von der Emde, 2001). As such, shape would be an important cue to be able to extract from objects to improve their identification and subsequent categorization.

**Figure 2:**
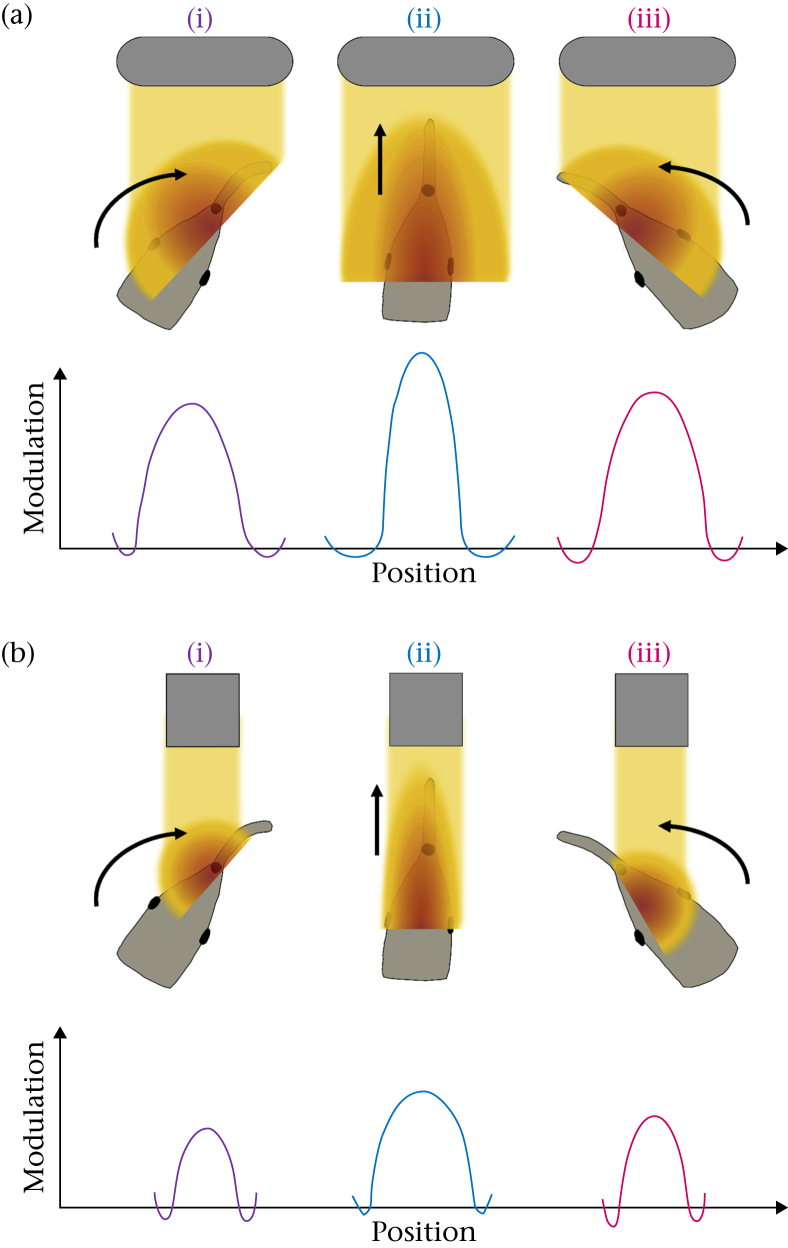
Schematic illustrating how *G. petersii* might use movement-induced modulations (MIMs) to encode an object's shape. Diagrams i–iii show an individual moving its head forwards while investigating either (a) a sausage-shaped object or (b) a cube in front of it (as in our experiments), and how the electric image might shift as a result. The fish approaches (i) from the left, (ii) straight on and (iii) from the right. The yellow-red zones are the electric images projected onto the skin by the objects and denote areas of changing electrical activity they induce. The darker (red) areas indicate areas experiencing increases in local amplitude, and the paler (yellow) areas indicate areas experiencing decreases in local amplitude. Below each fish, a hypothesized profile of the electric image (with its characteristic ‘Mexican hat’ shape, see Caputi et al., 1998) has been drawn to show what might happen at each action point. We propose that the magnitude and nature of the electric images generated will be influenced by an object's shape as indicated here.

Instead of using static electric images to determine the shape of an object, *G. petersii* might use the temporal series of electric images (electric flow) generated by engaging in movements around the object (Caputi & Budelli, 2006; von der Emde, 2006; Hofmann, Sanguinetti-Scheck, Künzel, et al., 2013). These movements will change the spatiotemporal dynamics of the electric field, and by analysing the changes in the electric field over successive electric images, they might be able to extract the necessary information required for shape recognition (Schumacher, Burt de Perera, & von der Emde, 2016; Fujita & Kashimori, 2019; Fig. 2).

It is already well documented that weakly electric fish like *G. petersii* will exhibit multiple stereotyped behaviours (‘probing motor acts or PMAs’) when exploring objects (see Toerring & Belbenoit, 1979; Toerring & Moller, 1984; von der Emde, 1992; von der Emde & Fetz, 2007; reviewed by Skeels et al., 2023a). These behaviours can range from swimming back-and-forth along an object (va-et-vient) to circling an object (lateral probing) at set distances (Toerring & Belbenoit, 1979; Toerring & Moller, 1984). PMAs are thought to be associated with active electrolocation, given that they coincide with changes in signalling activity, and these behaviours will reduce or stop altogether when an individual is ‘electrically silenced’ by cutting the spinal cord anterior to the electric organ (Toerring & Belbenoit, 1979; Toerring & Moller, 1984). PMAs are thought to either position a fish in a way in which it optimizes active electrolocation (Toerring & Moller, 1984; von der Emde, 1992) or maximize the change in current flowing across their receptive surface (skin) to facilitate extraction of particular object properties (Toerring & Moller, 1984; see also discussion in Skeels et al., 2023a).

Recent work has shown these fish can use the electric flow created through movement as a cue for continuous distance estimation (Hofmann et al., 2017). Schumacher, Burt de Perera, and von der Emde (2016) proposed that movement-induced modulations (MIMs) might act as a cue for shape. MIMs refer to the ‘[temporal] changes in the electrical images that occur as a fish swims past an object’ (Schumacher, Burt de Perera, & von der Emde, 2016). They theorized that the magnitude and nature of these modulations would be dependent on the object's shape and could provide a suitable cue for three-dimensional shape detection (Schumacher, Burt de Perera, & von der Emde, 2016; Fig. 2).

To test for MIMs, Schumacher, Burt de Perera, and von der Emde (2016) trained fish to discriminate between objects that either generated similar or different levels of MIMs (other variables were controlled for). They found individuals could discriminate the objects up to further distances when there was a large difference in MIMs, in other words when the objects appeared most different. Although Schumacher, Burt de Perera, and von der Emde (2016) found evidence to suggest a possible role of MIMs in shape recognition and discrimination, their limited sample size of two fish and inability to discount nonmovement cues prevented them from drawing firm conclusions.

Our study aimed to test the role of MIMs in shape recognition by manipulating the motor component of fish behaviour and testing whether movements (like the PMAs described above) are important for shape acquisition. To do this, we trained six fish in a two-alternative forced-choice (2AFC) set-up to discriminate between two objects differing only in shape. Once the fish learned the task, we varied the space available for them to undertake scanning movements next to the objects, assuming that this negatively altered the amount of MIMs occurring, and tested whether this led to a change in discrimination performance. If MIMs were involved in shape discrimination, we would expect an individual's discrimination performance to decline as swimming space, and thus MIMs, were restricted. Our study is therefore essential for determining the significance of body movements for three-dimensional shape recognition during active electrolocation in these fish.

## Methods

### Subjects

We trained six *G. petersii* of unknown sex and age to complete a shape discrimination task in a 2AFC set-up. We sourced our fish from a licensed fish dealer (The Goldfish Bowl, Oxford, U.K.). They had a total length of ca. 13.1–16.2 cm (including their chin appendage, the Schnauzenorgan). Each fish was housed in a standard 2-foot tank (ca. 60.2 × 35.2 cm and 31.5 cm deep) which also served as its experimental space. Reverse osmosis water was treated with a remineralization formula to ensure fish wellbeing and stabilization of the aquarium systems (Tropic Marin, Tropical Marine Centre, Chorleywood, U.K.). Water conductivity was 415 ± 35 μS/cm, water temperature was 25.5 ± 0.5 °C and pH was 7.3 ± 0.3. We set the lights to a 12:12 h light:dark cycle. Experiments were conducted during the light portion (20–30 lx). Fish were fed bloodworms (Gamma, Tropical Marine Centre, U.K.). They were only fed during training trials when participating in the study. Outside experiments, fish were given bloodworms twice a day, six times a week. We also supplemented their diet with brine shrimp (twice weekly) and additional vitamins (once a week). We provided plenty of enrichment (e.g. caves, tunnels and plastic ornaments) to keep them engaged.

### Ethical Note

This study was approved by the Department of Biology's ethics committee and conducted in accordance with the University of Oxford's animal ethics guidelines. No specific licences were required as the work undertaken was noninvasive. We took steps to ensure the wellbeing of our animals (see above) and limited the number of individuals kept in line with the ASAB/ABS Guidelines (ASAB Ethical Committee & ABS Animal Care Committee, 2018). For example, we chose a light level (20–30 lx) that was within the natural range of *G. petersii.* Our lights were specially programmed to slowly ramp up in intensity at the start of the light cycle and ramp down slowly at the end of the light cycle (on the time frame of hours) to ensure that there were no sudden changes in light intensity that could frighten the fish. We completed experiments when the light was most stable (i.e. in the middle of the light cycle). We used visual indicators of stress to monitor wellbeing throughout, such as skin colour changes, increased ventilation of gills and changes in swimming activity (see below and review by Huntingford et al., 2006). Our fish rarely showed these signs of stress, but when they did, the experiment was stopped immediately so that they could recover. Affected trials were omitted from our analysis. After this study, the fish participated in another related study and were then rehomed.

### Light

In this study, it was not necessary to exclude visual cues by completing the experiment in the dark. Previous studies have shown robustly that *G. petersii* have a very strong preference for using their active electric sense to recognize objects at closer distances (Schumacher, Burt de Perera, Thenert, & von der Emde, 2016; Schumacher, Burt de Perera, & von der Emde, 2017; Schumacher, von der Emde, & Burt de Perera, 2017). This strong dominance of the electric sense is called electrosensory capture (Schumacher, Burt de Perera, & von der Emde, 2017). Even when they have access to visual information, they do not use it for recognition of nearby objects (Schumacher, Burt de Perera, & von der Emde, 2017; Schumacher, von der Emde, & Burt de Perera, 2017; von der Emde & Zeymer, 2020). In fact, it is extremely difficult to get *G. petersii* to use visual cues instead of electric cues during object recognition tasks (Schumacher, Burt de Perera, Thenert, & von der Emde, 2016; Schumacher, Burt de Perera, & von der Emde, 2017; Schumacher, von der Emde, & Burt de Perera, 2017). This is due to their eyes having very poor spatial resolution, with the smallest discrimination angle being 3 degrees (Francke et al., 2014; Kreysing et al., 2012; Landsberger et al., 2008; Pusch, Kassing, et al., 2013; Pusch, Wagner, et al., 2013). Instead, their visual system is specialized for the detection of objects at distance, such as fast-moving predators in a current (Francke et al., 2014; Kreysing et al., 2012; Landsberger et al., 2008; Pusch, Kassing, et al., 2013; Pusch, Wagner, et al., 2013) or landmarks for longer-range navigation (Schumacher, von der Emde, & Burt de Perera, 2017). Many recognition studies with this species have shown that when objects were nearby, conducting experiments in the light yielded exactly the same results as when the light was turned off (e.g. von der Emde, 2004; von der Emde & Fetz, 2007; von der Emde et al., 2010; Fechler et al., 2012; reviewed by von der Emde & Zeymer, 2020). Given this, we feel it is unlikely that our fish were using visual cues during object discrimination in our experiments, but instead were primarily using their active electric sense. The behaviour of these fish (movement of body and Schnauzenorgan, i.e. engagement in PMAs) also strongly suggests that they were undertaking active electrolocation (see Results; Toerring & Belbenoit, 1979; Toerring & Moller, 1984; von der Emde, 1992; von der Emde & Fetz, 2007).

### Sample Size

We trained six individuals which is similar in number to those used in previous experiments on object recognition in *G. petersii* (e.g. von der Emde & Fetz, 2007; Fechler & von der Emde, 2013). We wanted to limit the numbers of individuals kept in captivity while ensuring we had a sufficient sample size to test our hypothesis robustly (ASAB Ethical Committee & ABS Animal Care Committee, 2018). In our study, we were interested in examining the principal effects of movement restriction on shape recognition rather than general population effects. In other words, we wanted to test whether *G. petersii* could use movement to aid shape recognition, rather than how widespread this ability might exist in wild populations. Our power analysis confirmed that six individuals were sufficient to test this question robustly (see Results for further details).

### Experimental Set-up

**Figure 3:**
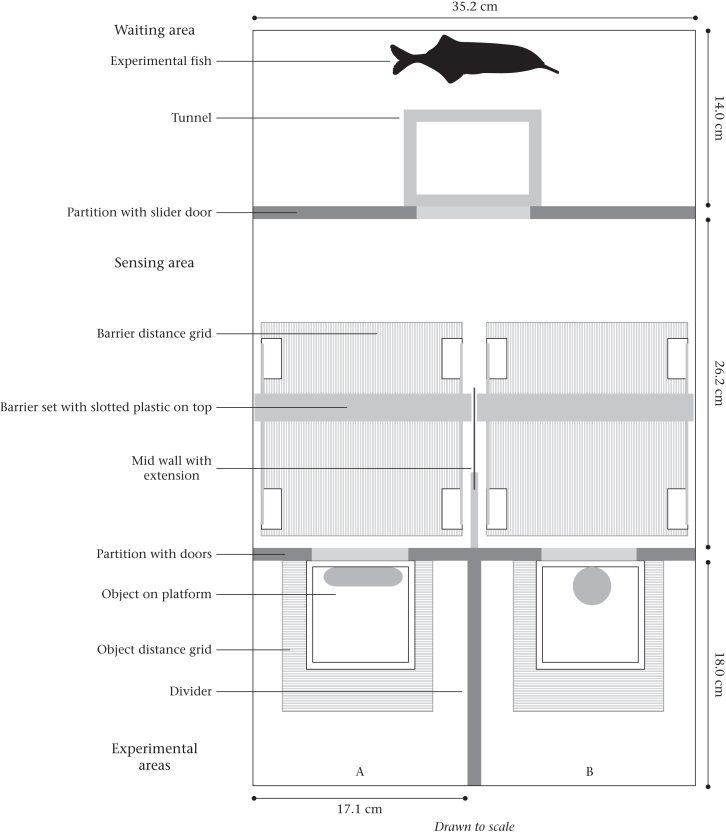
Top view of the experimental two-alternative forced-choice set-up used during shape discrimination trials.

**Figure 4:**
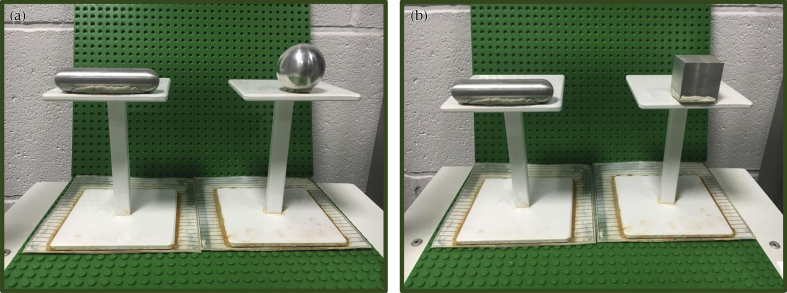
Objects used in the shape discrimination task. (a) Fish in group 1 (*N*=3) were trained to discriminate between a sausage-shaped object (S+) and a sphere (S-). (b) Fish in group 2 (*N*=3) were trained to discriminate between a sausage-shaped object (S+) and a cube (S-). Objects varied only in their shape (material, volume and distance were controlled for).

Each tank was made up of four compartments: the waiting area (14.0 × 35.2 cm) where the fish began trials, the sensing area (26.2 × 35.2 cm) where the fish decided which object to choose, and the two experimental areas, A and B (each 18.0 × 17.1 cm) which contained either the positive or negative object (Figure 3, Figure 4). Outside of experiments, the fish could access all areas of their tank, but these now contained normal enrichment rather than experimental apparatus.

The partitions, barriers and doors all had nylon mesh windows (mesh size: 0.71 mm per square) which meant that water could flow through them, ensuring that they did not form barriers for active electrolocation (Schumacher, Burt de Perera, & von der Emde, 2016). Barriers were placed in the sensing area to limit the amount of space that the fish could occupy and use for scanning movements during active electrolocation (Fig. 3). Two barriers were placed either side of the opening to A, and the same for B (Fig. 3). The overall size of each barrier was 18.4 × 14.4 cm and 0.2 cm deep, and the mesh window was 14.8 × 7.9 cm. For training, the distance between each barrier pair was always 15.9 cm (D1), providing the maximal possible space. For testing, the distance was varied to one of four distances (see Testing regime).

### Pretraining

These sessions ensured the six fish were familiar with the set-up before training fully commenced. They learned to swim out of the waiting area and into the sensing area when the slider door was opened. A clear tunnel (28.9 × 10.9 cm and 7.7 cm deep) was placed at the entrance of this door to ensure the fish did not leave the waiting area at too much of an angle in case this had a bearing on choice (Fig. 3). They then learned to swim through the channels made by the barriers, open the doors leading to A and B (using their Schnauzenorgan), enter A/B to get a reward and then return to the waiting area. They were also given time to acclimatize to the object platforms.

### Experimental Objects

The fish were split equally into two training groups. All fish were trained with the same positive object (S+) but each group was provided with a different negative object (S−). We did this to see whether object pairing had an impact on performance. S+ was a sausage-shaped object (ca. 1.5 × 6.3 cm and 1.5 cm high). S− was either a cube (side length 2.42 cm) or sphere (diameter 3 cm). The sausage-shaped object was presented with its longest side facing the door and the cube was presented with one of its sides facing the door (Fig. 4). All objects were made from the same material (aluminium) and were of similar volumes, meaning that these properties should not have impacted choice (von der Emde & Fetz, 2007).

Each object was positioned on a platform (overall height ca. 10.8 cm). Objects were placed ca. 1.6 cm from the openings to A and B if fish were trained without the doors and ca. 1.9–2.5 cm away if trained with the doors (we had to account for door depth and water flow pushing doors out slightly here). We trained and tested two individuals with the doors removed to check that the doors themselves did not influence performance (fish 5 and 6).

### Training Regime

The fish were trained to swim into the area with S+ (which was associated with a food reward, a bloodworm) and to avoid the area with S− (which was associated with a mild punishment, a gentle tap of the glass followed by being shooed back to the waiting area). This combination of sound and movement was aversive to the fish but not enough to inflict stress. We used visual indicators of stress to monitor wellbeing throughout, such as those described in Huntingford et al. (2006). *Gnathonemus petersii* will increase ventilation of their gills, turn pale and ‘freeze’ on the spot when they appear stressed (Amey-Özel et al., 2015; Toerring & Moller, 1984). Our fish rarely exhibited these signs of stress, but when they did, we stopped experiments immediately so that they could recover. Affected trials (*N* = 3) were omitted from analyses.

The position of S+ was randomized to prevent choice being determined by object location. Generally, each fish completed 24 trials in a session. One session was conducted with each fish per day. A total of 3214 training trials were conducted across all fish during the training phase (fish 1 = 312, fish 2 = 480, fish 3 = 264, fish 4 = 655, fish 5 = 629 and fish 6 = 874 trials). An Apeman A70 Action Camera recorded the behaviour of the fish during all trials.

### Testing Regime

**Fig. A1:**
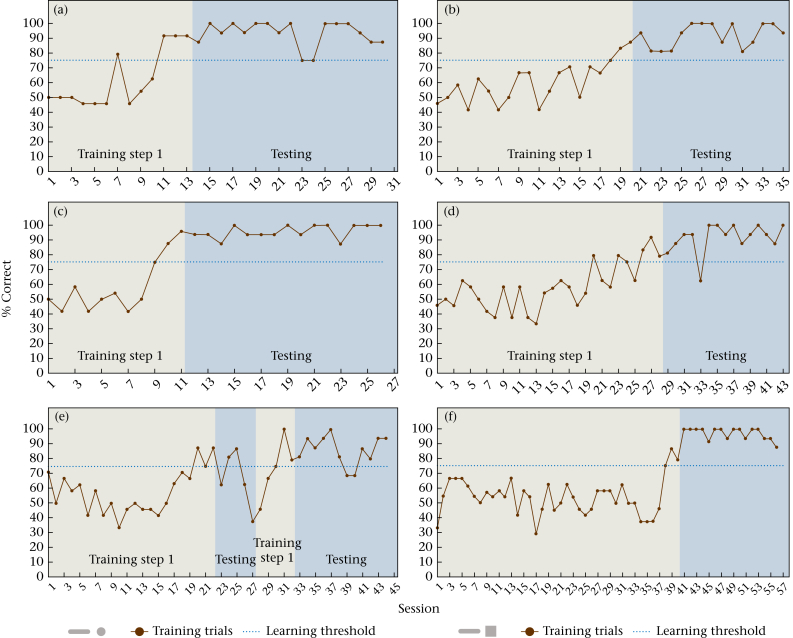
(a–f) Learning curves for fish 1–6. Fish were trained to discriminate between two differently shaped objects. S+ (a sausage-shaped object) was the same for all fish. Fish 1 (a), 3 (c) and 5 (e) had the sphere as their S-, whereas fish 2 (b), 4 (d) and 6 (f) had the cube. Fish 1–4 were trained with doors leading to the experimental areas (A and B) where the objects were placed, whereas fish 5 and 6 were not. During training step 1, an individual was trained to distinguish between S+ and its S−. Once the fish had reached or exceeded the learning threshold of 75% on three consecutive sessions (dotted line) they moved onto testing. During testing, training trials were interspliced with test probes to maintain motivation and performance. The scores from these training trials are plotted here. Total *N* = 4722 training trials across both experimental phases (fish 1 = 584, fish 2 = 720, fish 3 = 504, fish 4 = 895, fish 5 = 893 and fish 6 = 1126).

Testing commenced once individuals had reached the preassigned learning criterion of 75% correct over three consecutive training sessions (as described in Schumacher, Burt de Perera, Thenert, & von der Emde, 2016; Schumacher, Burt de Perera, & von der Emde, 2016; see Appendix Fig. A1). Test trials were introduced every third trial. During these trials, the distance between the barriers was changed. The barriers could be placed at four possible positions: 15.9 (D1), 12.7 (D2), 9.5 (D3) or 6.3 cm apart (D4). Fish were neither rewarded nor punished to prevent further learning (von der Emde & Fetz, 2007; Schumacher, Burt de Perera, & von der Emde, 2016; Schumacher, Burt de Perera, & von der Emde, 2017). However, testing was interspliced with training trials (two for every test trial) to maintain discrimination performance and keep motivation high (von der Emde & Fetz, 2007). On average, we conducted 30 test trials at each distance for every individual studied. In total, we conducted 1460 training trials (fish 1 = 240, fish 2 = 240, fish 3 = 240, fish 4 = 240, fish 5 = 248 and fish 6 = 252 trials) and 720 test trials (fish 1 = 118, fish 2 = 118, fish 3 = 120, fish 4 = 117, fish 5 = 123 and fish 6 = 124 trials) in the testing phase alone.

### Post Hoc Analyses

#### Latency of response during test probes

Latency was defined as the time it took an individual to choose an object (measured from when the slider door was opened to when the fish had made its choice by entering either A or B completely). We were interested in looking at latency as a proxy for certainty. If individuals were using MIMs, we expected them to be more uncertain (and so take longer to make a choice) with the narrowing of the barriers, as scanning movements would be less effective in extracting shape information, and so more time inspecting the objects would be needed.

#### Side-switching behaviour during test probes

Side switching was defined as the number of times an individual went between the two sides of the sensing area before making a choice and was examined as another proxy for certainty. If individuals were using MIMs, we expected them to be more uncertain (and so switch between the two sides more) when the barrier distance decreased, as scanning movements would be restricted, and so more inspections would be needed to discern the objects.

### Statistics

#### Shape discrimination performance

We used binomial generalized linear mixed models (GLMMs) to test for an effect of barrier distance on shape discrimination performance. Our main model contained two fixed effects (barrier distance and object pairing) and one random effect (fish ID). Barrier distance was considered as a continuous variable as distance scales, whereas object pairing and fish ID were treated as categorical variables. We investigated this model further to determine what the effect size of distance might be (see Appendix for more details). This analysis was run in RStudio, R version 3.6.2 (RStudio Team, 2022) using the ‘lme4’ package (Bates et al., 2015).

We also ran a post hoc analysis to compare discrimination performance at the different barrier distances. We used the same model as above, but this time, barrier distance was treated as a categorical variable (see Appendix for further details). This analysis was run in RStudio, R version 4.1.2 (RStudio Team, 2022), using the glht function in the ‘multcomp’ package (Hothorn et al., 2008) and *P* values were adjusted using the Holm–Bonferroni method.

#### Latency of response during test probes

We used gamma GLMMs to determine whether barrier distance had a significant effect on latency. Our main model had two fixed effects, barrier distance and trial outcome (which we hypothesized would be correlated with level of certainty), with the random effect set as fish ID. Barrier distance was considered as a continuous variable, whereas trial outcome and fish ID were treated as categorical variables (see Appendix for more information). This analysis was run in RStudio, R version 3.6.2 (RStudio Team, 2022) using the ‘lme4’ package (Bates et al., 2015).

#### Side switching during test probes

We used Poisson GLMMs to determine whether barrier distance had a significant effect on side switching (visits were coded as counts, such as 0, 1, 2, 3 … etc). Our main model stated that side switching depended on barrier distance and trial outcome (fixed effects) and fish ID (random effect). Again, barrier distance was considered as a continuous variable, whereas trial outcome and fish ID were treated as categorical variables (see Appendix for more details). This analysis was run in RStudio, R version 4.1.2 (RStudio Team, 2022) using the ‘lme4’ package (Bates et al., 2015).

#### Sample size

A power test was performed post hoc to verify and confirm that the number of individuals tested was sufficient to produce enough statistical power to examine the change in discrimination performance across barrier distances.

The power achieved to test the effect of changing barrier distance on shape discrimination performance with six individuals and 80 test trials was above the 80% threshold criterion (power for 1000 iterations of the GLMM = 100.00%, 95% confidence interval [99.63, 100.00]). This indicates that the number of fish we had (*N* = 6) and the number of test trials with each (ca. 120) would have provided us with sufficient statistical power. This analysis was run in RStudio, R version 4.1.2 (RStudio Team, 2022) using the ‘simr’ package (Green & MacLeod, 2016).

## Results

### Shape Discrimination Performance

**Figure 5:**
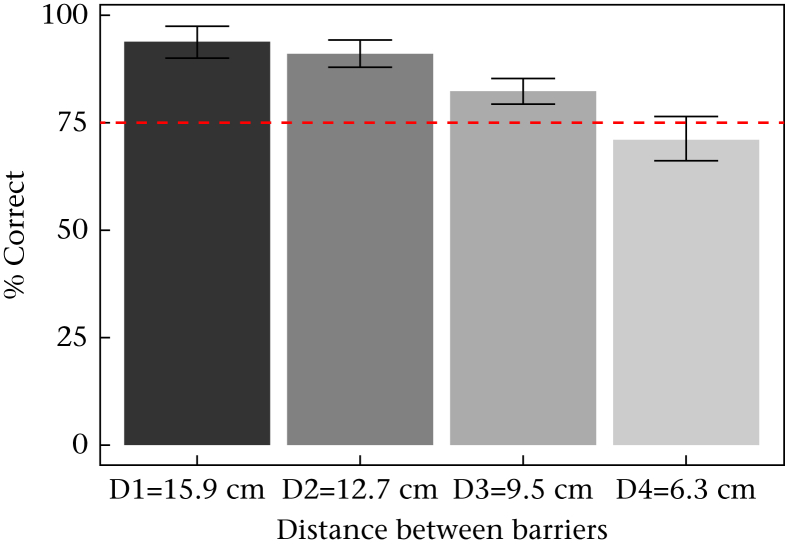
Group mean discrimination performance (with SE bars) during the testing phase. Performance was measured as the percentage of trials that were correct for a given distance (D1–D4). The dashed line denotes the learning threshold of 75% correct. Total number of trials = 720 (for an individual breakdown see Testing regime in the Methods).

**Fig. A2:**
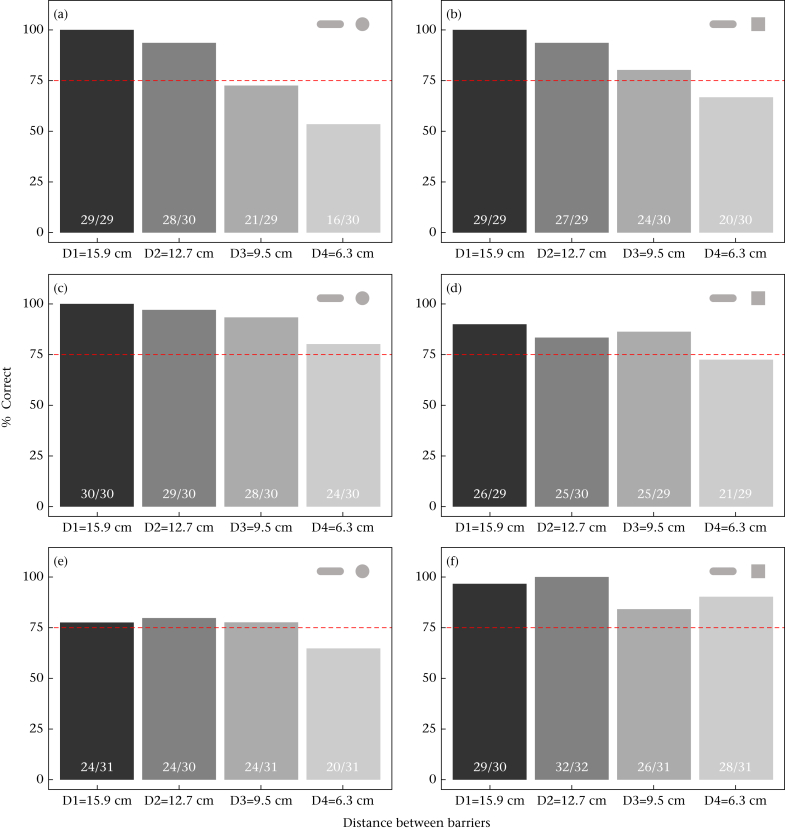
(a–f) Individual discrimination performance during the testing phase. Fish 1 (a), 3 (c) and 5 (e) had the sphere as their S−, whereas fish 2 (b), 4 (d) and 6 (f) had the cube. The numbers within each bar show how many trials were correct against how many were run. The dotted line represents the learning threshold of 75%. Note that fish 5 (e) and 6 (f) were trained and tested without the doors leading to the experimental areas (A and B) where the objects were placed.

We found that an individual's ability to discriminate differently shaped objects was significantly influenced by how far apart the barriers in the sensing area were (GLMM: *Z*_716_ = −6.184, *P* < 0.001; Fig. 5, A2; see effect size information in Tables A1, A2, A3). All fish learned to discriminate between the two objects offered (Fig. A1). However, they generally found it harder to correctly identify the positive object as the distance between the barriers was reduced (Fig. 5, A2; Table A1). This was regardless of which objects the fish were trained and tested with (GLMM: *Z*_716_ = 0.795, *P* = 0.426). Door presence/absence did not seem to influence discrimination performance (Fig. A2).

Our pairwise comparison detected significant differences in discrimination performance between all barrier distance combinations, except for D1 and D2 (Table A4). The most notable differences were observed between the wider barrier distances, D1 and D2, and the shortest barrier distance, D4 (Table A4).

### Notable Behaviours During Testing Phase

During training and test probe trials, *G. petersii* displayed particular behaviours while investigating the objects behind doors A and B. When leaving the waiting area, individuals tended to head to one side first, often the side that they had a pre-existing preference for. This preference seemed to be individual specific and likely developed over time. During their approach, the head, Schnauzenorgan and body were held out relatively straight and level in the water column. Once beside the objects, they adjusted their body and head positions frequently and were often more angled. We observed several types of PMAs during trials (Toerring & Belbenoit, 1979; Toerring & Moller, 1984; von der Emde & Fetz, 2007). Head/stationary probing and chin probing were observed when the fish were close to the objects. Tail probing was observed when the fish were unfamiliar with an object or seemed wary of it. Tangential probing occurred when the fish examined one object before rapidly changing direction to explore the other object. Whole-body back-and-forth (va-et-vient) motions were observed less than other motor acts due to the constraints on space. When barriers were at their most restrictive (D4), fish were less able to perform PMAs in front of the objects and movements were more limited except for some back-and-forth movements. They also adjusted their door-opening technique. Fish trained without the doors also showed similar shifts in the way they used the space around the objects.

### Post Hoc Analyses

#### Latency of response during test probes

**Figure 6:**
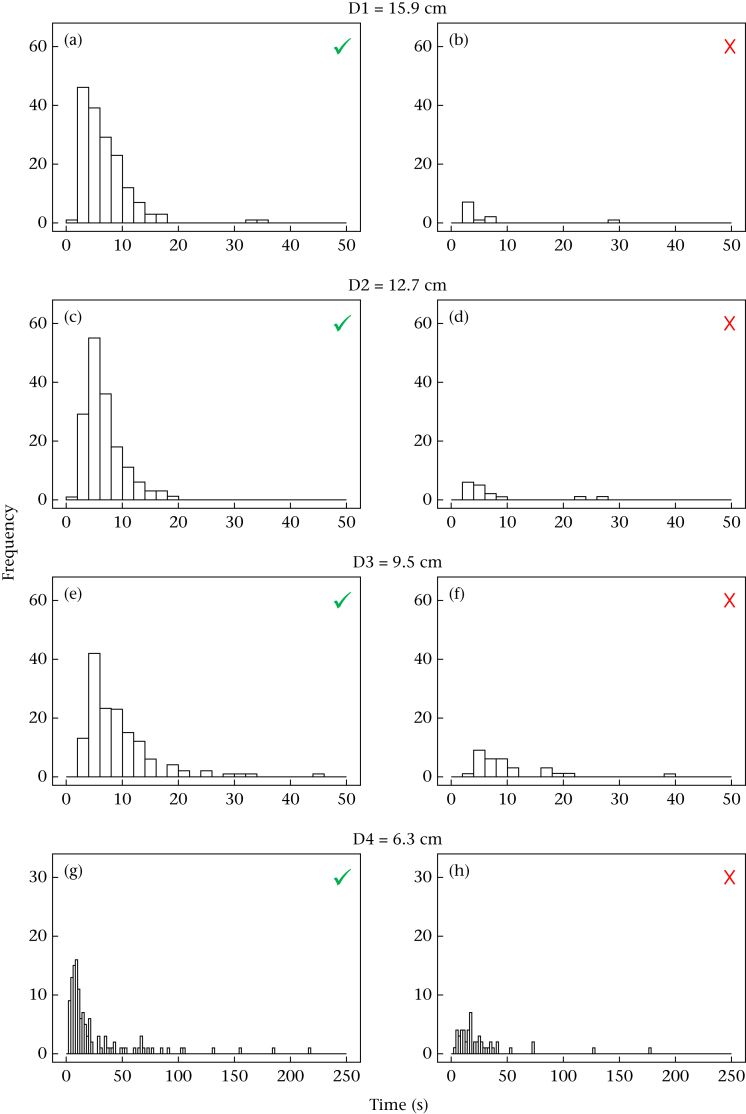
(a–h) Response times for completing the shape discrimination task during test probe trials at distances D1–D4. Each data point represents the time (s) it took an individual to make a choice in a single trial. Times have been pooled to show responses on a group level. For each distance, response times have been categorized according to whether the trial resulted in (a, c, e, g) a correct (tick) or (b, d, f, h) an incorrect (cross) choice being made. Note that the same *x*-axis range has been used for D1–D3, but a larger *x*-axis range was required for D4 due to the steep increase in response times. Bin size was the same for all conditions to aid comparisons. *N* = 708 trials (*a* = 165, *b* = 11, *c* = 163, *d* = 16, *e* = 146, *f* = 31, *g* = 125 and *h* = 51).

We found that barrier distance was a significant predictor of latency (GLMM: *t*_703_ = −19.949, *P* < 0.001; Fig. 6). Latency increased as barrier distance decreased, with the most noticeable increase occurring at the narrowest distance, D4 (mean latency ± SE: *D*1 = 6.88 ± 0.36 s, *D*2 = 6.74 ± 0.28 s, *D*3 = 9.25 ± 0.48 s, *D*4 = 26.19 ± 2.60 s; Fig. 6). Trial outcome did not have a significant effect on latency (GLMM: *t*_703_ = 0.677, *P* = 0.499; Fig. 6).

#### Side-switching behaviour during test probes

**Figure 7:**
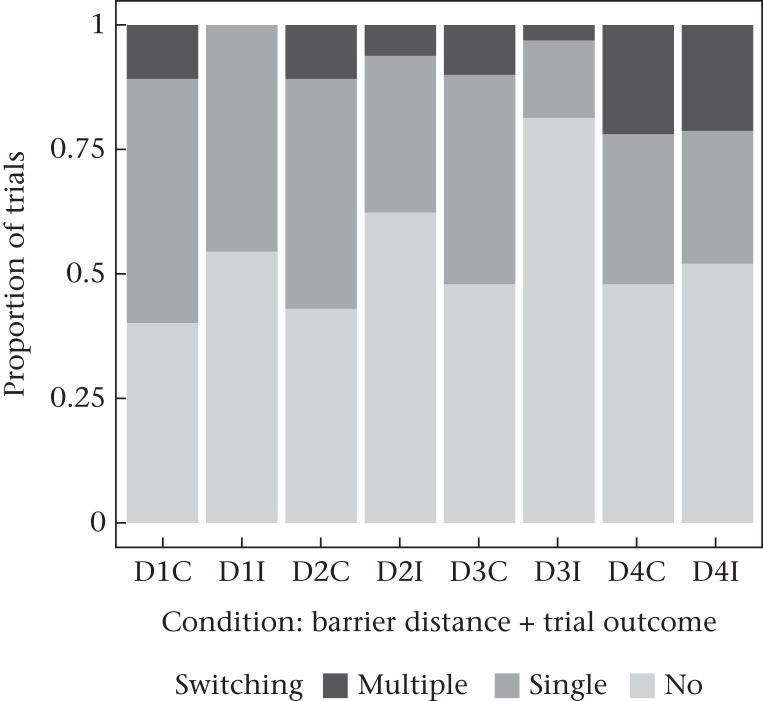
Group side-switching behaviour during test probe trials. Trials were placed into one of eight conditions. The condition was dependent on the barrier distance used for that trial (D1–D4) and the outcome of the trial (C=correct trial and I=incorrect trial). Greyscale bars indicate the type of side-switching behaviour observed (no switching, single instance, two or more instances). The size of each bar represents the proportion of trials that fitted each category. Total number of trials = 720 (for an individual breakdown see Appendix).

**Fig. A3:**
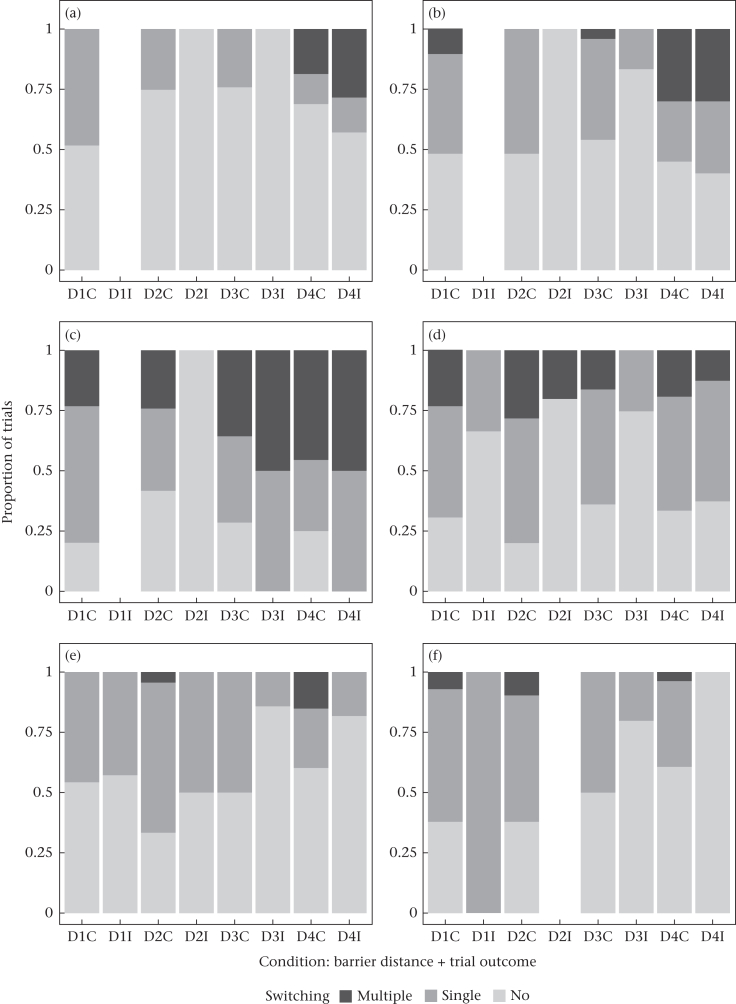
(a–f) Side-switching behaviour of fish 1–6 during test probe trials. Trials have been separated according to barrier distance being tested (D1, D2, D3 or D4) and trial outcome (C = correct or I = correct). Greyscale bars indicate the type of side-switching behaviour observed (no switching, single instance, two or more instances). The size of each bar represents the proportion of trials that fitted each condition. Blank spaces denote conditions where no trials were available to assess. Note that fish 5 (e) and 6 (f) were trained and tested without the doors leading to the experimental areas (A and B) where the objects were placed. Number of trials: fish 1=118 (a), fish 2=118 (b), fish 3=120 (c), fish 4=117 (d), fish 5=123 (e) and fish 6=124 (f).

Individuals tended to prefer to limit their side switching where possible, with most trials showing no side switching or only a single side switch (Fig. 7, A3). Nevertheless, we found that barrier distance had a significant influence on side-switching behaviour (GLMM: *Z*_716_ = 2.905, *P* = 0.004). More switching occurred as barrier distance was narrowed, particularly at the shortest distance, D4 (Fig. 7). The amount of switching varied across individuals (Fig. A3). Fish 5 and 6 showed less multiple switching compared to the other fish studied, and both were trained and tested without the doors (Fig. A3). Lastly, trial outcome did not have a significant influence on side switching (GLMM: *Z*_716_ = 0.725, *P* = 0.469; Fig. 7).

## Discussion

We investigated the role of egocentric movement in shape discrimination in *G. petersii.* We were interested in determining whether movement-induced modulations (MIMs) could be a useful method for extracting shape information. To determine this, we trained fish to complete a shape discrimination task in a 2AFC set-up. We manipulated the space available for scanning movements to determine whether this had an impact on their ability to distinguish the objects correctly.

### Shape Discrimination Performance

We found that barrier distance had a significant effect on an individual's ability to discriminate between the two objects (Fig. 5, A2, Table A1). Individuals found it more difficult to discriminate the objects as the barrier distance narrowed and space around the object became more limited, regardless of the objects they were trained and tested with (Fig. 5, A2, Table A1). This supports our hypothesis that *G. petersii* can use movement to facilitate the extraction of shape information from the environment.

We found that the biggest drops in discrimination performance tended to occur at the shortest barrier distances (Fig. 5, A2, Table A1). These drops likely represent a threshold being reached, where the amount of space available was not enough for fish to reliably scan the objects with the active electric sense, and so, lacking enough information on the objects’ shape, they more frequently made mistakes. This is also supported by our pairwise comparison which showed that performance levels differed significantly at all distance combinations (except between *D*1 and *D*2), and with the most noticeable differences occurring between the widest and narrowest barrier distances (e.g. *D*1–*D*4 and *D*2–*D*4; Table A4).

The hypothesis that the barriers shaped the movement of the fish is further supported by our observations at the shorter distances. We found individuals tended to change their movements when beside the objects, and this was seen across all fish, regardless of whether they were trained with the doors, suggesting the changes were due not to the presence or absence of doors but to the barriers themselves (see Results: Notable behaviours during testing phase).

Given that we did not block out any senses to avoid impacting behaviour, how can we be sure that our fish primarily used their active electric sense for shape discrimination and not another sense? In principle, an individual could get shape information about the two objects using vision (e.g. Schumacher, Burt de Perera, Thenert, & von der Emde, 2016). Since the two objects would have looked very different when observed from straight ahead, a reduction in space between the barriers should not have impaired visual discrimination. Nevertheless, it is very unlikely that vision was used in the first place, given the dominance of the active electric sense at the working range we were investigating (Schumacher, Burt de Perera, & von der Emde, 2017) and the fact that *G. petersii's* visual system is not well equipped to detect the fine details of an object at close range, but instead is adapted to detect large and fast-moving targets at a distance in poor optical (dimly lit and turbid) environments (Francke et al., 2014; Kreysing et al., 2012; Landsberger et al., 2008; Pusch, Kassing, et al., 2013; Pusch, Wagner, et al., 2013).

For electrical discrimination, however, movement was likely necessary. Since the objects had the same volume, they would have elicited relatively similar electric images when a fish swam straight ahead towards them without left and right movement (Fujita & Kashimori, 2019). Only when the fish was moving in front of the objects would the electric image have changed significantly because the greatest modulations would occur when the fish was close to the objects (Fig. 2). The nature of the modulations would have been dependent on the angle between the fish and the object itself (Hofmann, Sanguinetti-Scheck, Gómez-Sena, & Engelmann, 2013; Hofmann, Sanguinetti-Scheck, Künzel, et al., 2013; Fig. 2). The electric image would have also moved across the skin of the fish until the objects had been electrolocated from the sides (Fig. 2). These temporal changes of electric images and their movement on the skin of the fish might have been necessary to get enough information about an object's shape (Hofmann, Sanguinetti-Scheck, Gómez-Sena, & Engelmann, 2013; Hofmann, Sanguinetti-Scheck, Künzel, et al., 2013; Schumacher, Burt de Perera, & von der Emde, 2016; Fujita & Kashimori, 2019; Fig. 2).

For example, a sausage-shaped object and a cube would appear very distinct when inspected from different angles; Fig. 2. Owing to edge current effects, we would expect the more curved (sausage-shaped) object to project an electric image with a steeper slope on a curved surface (i.e. the front of the head) than on a flatter surface (i.e. the side of the head; see Pusch et al., 2008 for more information). As the viewpoint changes, so does the information being obtained (Fig. 2). Therefore, by engaging in many different movements around the object, like the PMAs described, the viewpoint will change and so will the electric image generated. This will be dependent on the object's geometry. For instance, the sausage-shaped object will project a large and elongated electric image, and the cube will project a smaller, rounder electric image onto the skin (Fig. 2). The corners of the cube would be associated with areas of higher amplitude and the edges with areas of lower amplitude. The head is not a flat surface, so the electric image will bend around and project onto the sides of the face (Fig. 2). Engaging in movements at different speeds and distances from the object will provide more information about an object's shape. This will be determined by how the images change, both spatially and temporally (with each object having its own MIMs signature). The movement is therefore necessary to help resolve object ambiguities which would normally exist when examining an object from a single perspective. Note that the magnitude of the modulations will also be affected by other properties, for example the location onto which the objects are projected. Stronger modulations will occur at the head and Schnauzenorgan due to the funnelling and tip effects experienced there (Pusch et al., 2008) and the high density of receptors present (Hollmann et al., 2008). Therefore, individuals tend to examine objects head on so that they can focus the objects onto the areas with most sensitivity, the so-called foveal regions (von der Emde et al., 2008). However, these location effects would not prevent an individual from being able to extract shape information from an object, as what matters is the activation patterns of receptors over space and time generated through movement at different distances, angles and speeds past the object (Hofmann, Sanguinetti-Scheck, Gómez-Sena, & Engelmann, 2013; Hofmann, Sanguinetti-Scheck, Künzel, et al., 2013).

If we impair movement of an individual, it might only get straight-ahead electric images, which do not change temporally and do not move on the skin (or move less), and therefore may not provide enough information for the fish to reliably discriminate between the two objects, especially if they are similar in shape. Given our findings that discrimination performance declined with barrier distance, and the dominance of the active electric sense for object recognition at the distances we tested (e.g. Schumacher, Burt de Perera, & von der Emde, 2017), we can be confident that these fish were primarily relying on their active electric sense when completing our shape recognition task. This is further supported by the fact that we observed several types of PMAs during trials (see Results). These movements are involved in active electrolocation (Toerring & Belbenoit, 1979; Toerring & Moller, 1984; von der Emde, 1992; von der & Fetz, 2007). They are thought to either focus the object onto the regions on the head with maximum sensitivity, the Schnauzenorgan and nasal region (Toerring & Moller, 1984; von der Emde et al., 2008) or create electric flow and thereby allow for the extraction of object information by comparing spatiotemporal patterns of activity (Hofmann, Sanguinetti-Scheck, Künzel, et al., 2013; Toerring & Moller, 1984).

Through mathematical modelling, Fujita and Kashimori (2019) found that the integrated effect of peak amplitude and half-maximum width of electric images provided the best measure of an object's shape, feasibly being extracted during exploratory swimming (Fujita & Kashimori, 2019). However, more research (i.e. testing with real fish) is required to validate this putative cue given that the model made several assumptions that do not hold true (objects were two-dimensional, body shape and skin conductivity were uniform, and the electric organ fired in a simple way across space and time; Fujita & Kashimori, 2019). The last three variables will vary according to the weakly electric species being studied. Given this, it is unknown whether *G. petersii* would be able to extract shape information via this mechanism.

In summary, we found that shape discrimination performance worsened as barrier distance declined, suggesting that individuals extracted shape information through movement, presumably from analysing successive electric images as they moved alongside the object, that is, via MIMs (von der Emde et al., 2010; Fechler & von der Emde, 2013; Hofmann, Sanguinetti-Scheck, Künzel, et al., 2013; Schumacher, Burt de Perera, & von der Emde, 2016; Fujita & Kashimori, 2019; Fig. 2). By engaging in movements at different angles, speeds and distances, individuals are expected to be able to get more information about an object and be able to recognize it better.

### Post hoc Analyses

#### Latency of response during test probes

We examined response times as an indicator of the level of certainty an individual experienced during trials under a set number of conditions. Latency increased as barrier distance decreased (Fig. 6), suggesting that individuals were more uncertain during trials at shorter distances, and so needed more time examining the objects before they could make a choice. This is likely because a discrimination threshold with MIMs was reached. This would have occurred when the space was restricted to such a degree that an individual was unable to scan the objects effectively with their active electric sense, meaning that extra scans were required to resolve any ambiguities that existed between the two objects. In other words, when the barriers were at their most restrictive, the fish were less successful at scanning the objects, so they tried harder (see below) and for a longer time.

#### Side-switching behaviour during test probes

We measured switching behaviours as another proxy of certainty. We expected individuals to perform fewer side switches (and take less time) when they were more certain of S+ but exhibit more side switches (and take more time) when they were less certain. Fish completed more side switches when the distance between the barriers narrowed (Fig. 7), supporting the hypothesis that the fish were primarily relying on MIMs to complete the shape recognition task, and that side switching was employed to add value in trials, for example to help resolve ambiguities between the two objects in situations of greater uncertainty through additional scans. In other words, by scanning more, individuals might have been able to get sufficient information to discriminate an object's shape, even if each scan was less effective in picking up this information. This is supported by von der Emde and Fetz (2007) who found that individuals that inspected both objects had greater success in picking S+ than those that examined just one object. As such, side switching likely played an important role in our study by minimizing the chance of the wrong object being chosen.

### Individual Variation

There was interindividual variation in test probe trial performances (Fig. A2). Interindividual variation is common in behavioural studies (e.g. Kolok, 1999). This might have been driven by a variety of factors including fish size, motivation and door set-up (Knudsen, 1975; Caputi & Budelli, 1995; Caputi et al., 1998; von der Emde, 1992; von der Emde & Fetz, 2007). However, the most likely explanation is that the variation arose from each fish approaching the task in its own preferred way. For example, fish 3 exhibited more side switching than any other fish. This might have resulted in more information being gathered about the two objects, resulting in a greater chance of identifying S+ correctly, thereby contributing towards their high performance levels (Figs. A2c, A3c). On the other hand, fish 6 took much longer than the others to learn the task (Fig. A1f), but once it did, it had an extremely high level of success (rarely dipping below 90%; Fig. A2f). This might be evidence of a slow but accurate learning strategy being employed (see review by Chittka et al., 2009 on speed–accuracy trade-offs in decision making). Even with this variation, our overall results were robust (Fig. 5, A2).

### Comparisons With Other Animals Using Different Sensory Modalities

Animals use movement in similar ways to enhance their perception of the environment regardless of their taxonomic group (e.g. Prescott et al., 2011) or preferred sensory modality (e.g. Hofmann, Sanguinetti-Scheck, Künzel, et al., 2013). For example, praying mantids cannot move their eyes and so they will undertake head peering (side-to-side) movements to estimate the distance of stationary objects (reviewed by Kral, 2012). This visual peering behaviour is also seen in mice (e.g. Parker et al., 2022), chickens, *Gallus gallus domesticus* (e.g. Stamp-Dawkins, 2002), and is well known in humans. Humans can recognize a three-dimensional object by changing their viewpoint and examining different sides of the object, which makes it easier to determine its depth (Tarr et al., 1998). These movements are similar to the back-and-forth movements we observe in weakly electric fish when examining objects (e.g. Toerring & Belbenoit, 1979). Indeed, Pedraja et al. (2018) recently showed that these movements enable distance estimation with the active electric sense via motion parallax. Echolocating bats can use movement to extract new information from the environment as well. For example, Yin and Müller (2019) found that some species can encode the direction of a target in the form of time–frequency Doppler signatures. Blind cave fish, *Astyanax fasciatus*, use the pressure waves generated through their swimming motion to locate objects that they can use as landmarks for navigation (Burt de Perera, 2004). These examples illustrate the importance of movement for sensory acquisition in a variety of animals using a diverse array of senses.

### Conclusions

Here, we have shown that movement helps *G. petersii* to recognize and successfully discriminate between objects differing in their shape. We found that performance worsened as the barrier distance was decreased. Our analysis supports the hypothesis that our fish were predominantly relying on their active electric sense for shape discrimination, and that movement-induced modulations (MIMs) of a series of electric images moving over the fish's skin generated by swimming was likely to be the cue being used. Although we cannot discount the possibility of nonmovement cues, it seems very unlikely they played a significant role, owing to the object controls we put in place and the manipulations of the motor space we made. This is supported by Fujita and Kashimori (2019) who found that single electric images provide ambiguous cues for shape recognition, with differently shaped objects producing similar Gaussian images. This also includes the integration of visual information, which is likely to have made only a minor contribution to object recognition (if at all) due to the dominance and specialism of the active electric sense for recognizing nearby objects and the weakness of the visual sense for capturing high spatial resolution information (Pusch, Wagner, et al., 2013; Schumacher, Burt de Perera, & von der Emde, 2017). Our study therefore contributes to the growing body of evidence that movement is essential for shaping sensory input in a wide range of taxa (see reviews by Land, 1999; Caputi, 2004; Nelson & MacIver, 2006; Mitchinson et al., 2011; Prescott et al., 2011; Hofmann, Sanguinetti-Scheck, Künzel, et al., 2013; Yang et al., 2016; Engelmann et al., 2021; Skeels et al., 2023a).

Our results and the examples given from other taxa indicate the fundamental importance of sensory–motor coupling to understanding animal behaviour generally. We suggest future studies might benefit from taking a comparative approach to interpret and understand these movements better. This could be done by examining the active sensing strategies of animals from different taxonomic groups or by taking a single group (e.g. mormyrid fish) and examining numerous species within it. This would allow researchers to draw up a more comprehensive list of similarities and differences between movements (e.g. How do these strategies work? How do they develop? What is their function(s)?). Taking an interdisciplinary approach is likely to be useful here to help address different elements of this problem (Skeels et al., 2023a). Ultimately, this would help us to determine how and why these behaviours might have evolved.

## Author Contributions

**Sarah Skeels:** Conceptualisation, Methodology, Investigation, Formal analysis, Resources, Data Curation, Writing – Original Draft, Writing – Review & Editing, Visualisation, Funding acquisition.

**Gerhard von der Emde:** Conceptualisation, Methodology, Resources, Writing - Review & Editing, Supervision.

**Theresa Burt de Perera:** Conceptualisation, Methodology, Resources, Writing - Review & Editing, Supervision, Funding acquisition

## Data Availability

Data are available as Supplementary Material.

## Declaration of Interest

None.

## Figures and Tables

**Table A1:** Predicted and observed values of success for a test trial given barrier distance

Distance	Predicted probability of success (%)		GLMM			Observed success (%)^1^	Predicted (observed) performance change (%)
	Estimate	95% CI	*Z*	*df*	*P*		
			−6.184	716	<0.001		
D1 (15.9 cm)	94.12	[84.28, 98.10]				93.82	
D2 (12.7 cm)	89.23	[69.08, 97.04]				91.16	D1–D2= −4.89 (−2.66)
D3 (9.5 cm)	81.07	[48.21, 95.41]				82.22	D2–D3= −8.16 (−8.94)
D4 (6.3 cm)	68.89	[27.94, 92.95]				71.27	D3–D4= −12.18 (−10.95)

*N* = 6 individuals, 720 observations (test probe trials).

1 Observed success was determined after pooling data from all individuals tested. For each barrier distance, success was then calculated as: (total number of correct trials/total number of trials run) × 100.

**Table A2:** Values for the estimate derived from the model that best described discrimination performance

Distance	Log odds ratio^1^	Odds ratio^2^	Likely to get correct^3^ (%)	Performance step change (%)
D0 (intercept)	3.433	30.983	96.87	
D1: 15.9 cm	2.774	16.020	94.12	D0-D1: −2.75%
D2: 12.7 cm	2.114	8.283	89.23	D1-D2: −4.89%
D3: 9.5 cm	1.455	4.283	81.07	D2-D3: −8.16%
D4: 6.3 cm	0.795	2.214	68.89	D3-D4: −12.18%

1 Log odds ratios were derived from values in the fixed-effect section of our model summary. We calculated them as follows for each distance: D0= intercept estimate only, D1 = parameter estimate + intercept, D2 = (2 × estimate) + intercept, D3 = (3 × estimate) + intercept and D4 = (4 × estimate) + intercept.

2 Odds ratios were calculated by exponentiation of the log odds ratios.

3 Percentage likely to get correct= (odds right/odds total (right + wrong)) × 100.

**Table A3:** Values for the lower and upper limits derived from the model that best described discrimination performance

Distance	Log odds ratio (for LL)	Odds ratio (for LL)	LL % likely to get correct	Log odds ratio (for UL)	Odds ratio (for UL)	UL % likely to get correct
D0 (intercept)	2.555	12.871	92.79	4.399	81.374	98.79
D1: 15.9 cm	1.679	5.363	84.28	3.944	51.634	98.10
D2: 12.7 cm	0.804	2.234	69.08	3.489	32.763	97.04
D3: 9.5 cm	−0.072	0.931	48.21	3.034	20.789	95.41
D4: 6.3 cm	−0.947	0.388	27.94	2.580	13.191	92.95

LL=lower limit; UL=upper limit. Values were calculated in the same way as in Table A2 but from the confidence intervals of the estimate.

**Table A4:** Effect of barrier distance on discrimination performance when barrier distance was considered as a categorical variable, with pairwise comparisons of performance at the different barrier distances

		Estimate	SE	*Z*	*P*
**Fixed effects**					
Intercept		2.651	0.424	6.256	<0.001
Distance (D2–D1)		−0.400	0.408	−0.981	0.326
Distance (D3–D1)		−1.219	0.369	−3.299	0.004
Distance (D4–D1)		−1.865	0.355	−5.254	<0.001
Distance (D3–D2)		−0.818	0.329	−2.487	0.038
Distance (D4–D2)		−1.465	0.313	−4.685	<0.001
Distance (D4–D3)		−0.646	0.259	−2.492	0.038
**Random effects**					
Fish (intercept)		σ^2^ = 0.212	σ = 0.461		

Barrier distance was treated as a categorical variable for the purposes of this analysis. *P* values were adjusted using the Holm–Bonferroni method.

## Appendix

### Shape Discrimination Performance During Testing

We used binomial GLMMs to test for an effect of barrier distance on shape discrimination performance. We initially created two models to predict correct object selection: the first contained two fixed effects (barrier distance and object pairing) and one random effect (fish ID), while the second dropped our main parameter of interest (barrier distance). We compared the models using a likelihood ratio test (LRT) to determine which one was a significantly better fit to our data. We found that our first model best fitted the data (LRT: *χ*^*2*^_1_=43.403, *P*<0.001). We then investigated this model to determine how barrier distance influenced the discrimination performance of our fish and what the effect size of distance was on performance.

### Latency of Response During Test Probes

We used gamma GLMMs to determine whether barrier distance had a significant effect on latency. We initially created two models. Our first model had two fixed effects: barrier distance (our key parameter) and trial outcome (we thought this would be correlated with level of certainty). Our random effect was fish ID. The second model omitted barrier distance. An LRT was then done to compare the models to assess which one best fitted our data. We found that model 1 best described the latency data (LRT: *χ*^*2*^_1_=378.79, *P*<0.001). This model was then chosen and investigated further.

### Side Switching During Test Probes

We used Poisson GLMMs to determine whether barrier distance had a significant effect on side switching. We created two models: our first stated that side switching depended on barrier distance and trial outcome (fixed effects) and fish ID (random effect) while the second omitted barrier distance as a predictor. An LRT compared the two models to assess which one was a better fit. We found that model 1 fitted the data better (LRT: *χ*^*2*^_1_=8.434, *P*=0.0037). This model was chosen and examined further.

### Random Effects

Our random effect was fish ID for all analyses. For the performance model, our random effect was *σ*^2^=0.212 and *σ*=0.460. For the latency model, our random effect was *σ*^2^= 0.001 and *σ*=0.024, and for the side-switching model, our random effect was *σ*^2^=0.115 and *σ*=0.340.
