# Supplementary material for: Weakly electric fish use self-generated motion to discriminate object shape
Source: Anim Behav. 2023 Nov;205:47–63. doi: 10.1016/j.anbehav.2023.08.002 (PMC13328067; doi:10.1016/j.anbehav.2023.08.002)
Supplement: Multimedia component 4 [file mmc4.docx]

**Key for latency data:**

**Fish/Individual:**

A=Fish 1

B=Fish 2

C=Fish 3

D=Fish 4

E=Fish 5

F=Fish 6

**Barrier_Distance:**

1=15.9 cm

2=12.7 cm

3=9.5 cm

4=6.3 cm

**Correct:**

0=Incorrect

1=Correct

**Latency_s:**

Given in seconds
